# Immunocompromised patients with persistent SARS-CoV-2 viral shedding ≥8 weeks, clinical outcomes, and virological dynamics: a retrospective multicenter cohort study, 2020–2024

**DOI:** 10.1128/aac.00658-25

**Published:** 2025-09-26

**Authors:** Clémentine de La Porte des Vaux, Nicolas Veyrenche, Kevin Da Silva, Nathalie Chavarot, Marianne Burgard, Olivier Paccoud, Florence Runyo, Margaux Garzaro, Claire Rouzaud, Alexandra Serris, Damien Vimpere, Dany Anglicheau, Luc Mouthon, Olivier Hermine, Marie-Anne Rameix-Welti, Fanny Lanternier, Olivier Lortholary, Cléa Melenotte

**Affiliations:** 1 Department of Infectious and Tropical Diseases, Hôpital Necker Enfants Malades, Assistance Publique des Hôpitaux de Paris (APHP), IHU Imagine, Université Paris Cité 555089, Paris, France; 2 Department of Virology, Hôpital Necker Enfants Malades, APHP, Université Paris Cité 555089, Paris, France; 3 National Reference Center for Respiratory Viral Infections, Institut Pasteur, Université Paris Cité 555089, Paris, France; 4 Department of Nephrology and Kidney Transplantation, European Hospital George Pompidou, APHP, Université Paris Cité 555089, Paris, France; 5 Intensive Care Unit, Hospital Necker-Enfants Malades, APHP, Université Paris Cité 555089, Paris, France; 6 Department of Nephrology and Kidney Transplantation, Hospital Necker-Enfants Malades, APHP, Université Paris Cité 555089, Paris, France; 7 Department of Internal Medicine, Hôpital Cochin, APHP, Université Paris Cité 555089, Paris, France; 8 Department of Hematology, Hospital Necker-Enfants Malades, APHP, Université Paris Cité 555089, Paris, France; IrsiCaixa Institut de Recerca de la Sida, Barcelona, Spain

**Keywords:** immunocompromised patients, persistent SARS-CoV-2 shedding, solid organ transplant (SOT), direct antivirals, viral resistance

## Abstract

Immunocompromised patients (ICPs) infected with SARS-CoV-2 are at higher risk of severe illness. Some experience persistent viral shedding beyond eight weeks, which is associated with increased mortality and invasive fungal infections. However, data on the clinical profile, treatment impact, and standardized management for these patients remain limited. We conducted a retrospective cohort study at Groupe Hospitalier Paris Centre between March 1, 2020, and February 10, 2024. We assessed symptomatic ICPs with persistent SARS-CoV-2 shedding (>8 weeks), analyzing clinical progression, time to viral clearance, and emergence of resistance mutations in relation to treatment regimens. Fifty-three patients were included: 53% were solid organ transplant (SOT) recipients, 42% had hematological malignancies (HMs), and 5% had other immunosuppressive conditions. Severe infections occurred in 32%, 91% required hospitalization, and 17% (*n* = 9) presented invasive mold infections. SOT recipients achieved clinical cure faster than HM patients (*P* < 0.01). Patients treated with direct antivirals showed significantly faster viral clearance (*P* = 0.03) than those treated with monoclonal antibodies (mAbs) or convalescent plasma. No resistance mutations emerged against remdesivir or nirmatrelvir/ritonavir. However, 54% of viral strains showed initial or acquired spike protein resistance to mAbs. Direct antiviral therapies, particularly remdesivir and nirmatrelvir/ritonavir, appear safe and effective in promoting faster viral clearance and clinical recovery in ICPs with persistent symptomatic SARS-CoV-2 infection.

## INTRODUCTION

The COVID-19 pandemic has now largely been controlled, thanks to the widespread use of COVID-19 vaccines, with incidence rates drastically decreasing worldwide over the winter of 2024–2025 (1). More than three and a half years after the pandemic, the virus continues to evolve genetically, with current variants now showing high transmissibility (RX) and resistance to all monoclonal antibody therapies (mAb) (2). The Omicron JN.1 variant and its descendants are currently dominant worldwide, with ongoing diversification into sub-lineages, including KP.3.1.1 or NB.1.8.1 (3). Immunocompromised patients (ICPs), representing nearly 3% to 6% of the general population in developed countries, remain at high risk for severe and persistent SARS-CoV-2 infections, which are associated with high mortality rates (4, 5). Prolonged SARS-CoV-2 viral shedding—lasting over eight weeks—has been linked to persistent symptoms, spike protein mutations, hospitalization, invasive aspergillosis, and an increased risk of death (6–11). Hence, clinicians continue to face difficulties in managing this immunocompromised population affected by SARS-CoV-2—including patients with persistent shedding—as there are no standardized, consensus-based treatment guidelines, due to the limited number of prospective studies on this population (12, 13). Persistent viral shedding can be observed after exposure to antiviral drugs that fail to eradicate the virus and could potentially induce resistance mutations and emergence of new variants (14–17). We present here a retrospective cohort study describing the clinical outcome, viral clearance, and virological characteristics, including the incidence of resistance-associated mutations that emerged during persistent SARS-CoV-2 viral shedding (>8 weeks) in ICPs according to the treatments they received.

## MATERIALS AND METHODS

### Study design and definitions

This retrospective cohort study was conducted at the Groupe Hospitalier Paris Centre and included immunocompromised adult patients with persistent SARS-CoV-2 viral shedding lasting over 56 days (>8 weeks) from March 1, 2020, to February 10, 2024.

### Patients definition and inclusion criteria

Persistent viral shedding was defined by at least two positive SARS-CoV-2 PCR tests (nasopharyngeal) over a period exceeding 8 weeks, with no negative tests or confirmed reinfections during this time (unless there was evidence of a positive repeat qPCR test within two days after a first negative nasopharyngeal qPCR, which was thus considered as a false negative). In the absence of a consensual definition of persistent viral shedding in immunocompromised patients, and in line with the definition recently proposed by Machkovech et al. (>30 days), we have chosen to work on an even more specific population with very prolonged persistent viral shedding >8 weeks, which often also presents numerous complications and therapeutic difficulties. This work is a continuation of a previously published study (6).

Included ICPs had conditions such as primary immune deficiencies, HIV with CD4 counts <200/mm³, autoimmune diseases, solid organ transplants (SOT), allogeneic hematopoietic stem cell transplants, chronic lymphoid malignancies, or were receiving immunosuppressive therapies. The management of COVID-19 patients within the Paris Centre hospital group was comparable and homogeneous in all the departments participating in this study. It followed the recommendations of the multidisciplinary consultation meetings. Asymptomatic patients without clinical data and those with reinfections were excluded. Severity was categorized based on the 2024 WHO criteria, defining severe infections as those requiring hospitalization with >3L oxygen and/or corticosteroids or tocilizumab (18).

### Data collection and outcome measures

The data collected included clinical and radiological presentations, SARS-CoV-2 variant, qPCR Ct values, underlying conditions, immunosuppressive treatments, and specific anti-COVID-19 therapies. Clinical and radiological outcomes were also collected, as well as kinetics of viral loads and sequencing after treatment (or during the time of infection in untreated patients) and the occurrence of complications: death, hospitalization, and probable or proven invasive mold infections (IMIs), according to EORTC/MSG definitions (18, 19). Outcomes included clinical cure (disappearance of symptoms), viral clearance (i.e., qPCR negativity), radiological resolution on CT scan, and complications, such as IMIs, hospitalization, and death. In the case of multiple treatments, two treatments were part of the same line if their initiation did not differ by more than five days.

### Sample preparation and sequencing

Sequencing analysis was performed for patients who had at least two available samples, spaced at least one week apart to ensure robust temporal data. In the case of treatment, we selected available samples before and after receiving anti-SARS-CoV-2 treatments. For optimal sequencing, among the samples collected, before and after treatment, we selected those with the lowest available Ct values in patients with cycle threshold (Ct) <28. Samples that were not exploitable due to poor sequencing results or poor-quality consensus were substituted by another available sample meeting optimal quality criteria. This selection process ensured reliable temporal consistency, treatment relevance, and data quality across the study cohort. Among the selected samples, nucleic acids were extracted using the NucleoSpin 8 virus Core Kit (Macherey-Nagel), followed by dual RT-PCR with LunaScript Supermix (NEB) and Q5 High-Fidelity DNA polymerase (NEB), using a pool of primers (ARTIC V5.3.2 from https://github.com/artic-network/primer-schemes/tree/master/nCoV-2019/V5.3.2). Purified amplicons were sequenced at the Mutualized Platform for Microbiology using the Nextera XT DNA Library Prep kit (Illumina) on the NextSeq 500/2000 systems (Illumina Inc.).

### Mutation and resistance analysis

For each sample, a consensus sequence was generated using an in-house bioinformatics pipeline. This pipeline includes read mapping with minimap2 (v2.26) and BWA (v0.7.17) and variant calling and consensus building with iVar (v1.3.1). The consensus sequence, reflecting the dominant viral population, was constructed by incorporating nucleotide positions with read frequencies above 60%. Minor variants below 40%, which can influence viral evolution and drug resistance, were also noted. For minor variants, a 5% frequency threshold was applied to exclude low-frequency noise. Mutations with read frequencies between 40% and 60% were marked with ambiguous nucleotides to account for intra-host diversity (20). Lineage assignment and phylogenetic annotation were performed using Nextclade (v3.8.2), following the global SARS-CoV-2 clade system and assessing sequence quality. A phylogenetic tree, with samples color-coded by patient and Pango lineage, was generated using multiple sequence alignment with MAFFT (v7.525), and phylogenies were inferred using IQ-TREE (v2.2.2.2) with the maximum likelihood and general time-reversible model. Drug-resistance mutations were screened in both consensus sequences and minor variants using the Stanford Coronavirus Antiviral & Resistance Database (updated 5/14/2024; https://covdb.stanford.edu/susceptibility-data/table-mab-susc/). Only mutations identified in at least one cohort sample were reported with their corresponding drugs.

### Statistical analysis

To compare the distribution of continuous and dichotomous variables between two groups, we used the χ^2^ test or the two-sided Fisher exact test, respectively. All tests were two-sided, and *P* < 0.05 was considered significant. Therapeutic impact (e.g., sotrovimab, nirmatrelvir/ritonavir, remdesivir, casirivimab/imdevimab, tixagévimab/cilgavimab, and plasmatherapy) was evaluated using χ² or Fisher’s exact tests for categorical data and log-rank tests for survival analyses, with significance set at *P* < 0.05. Analyses were performed with STATA v17.0.

## RESULTS

We included 53 ICPs with persistent viral shedding (>8 weeks) during the inclusion period. Omicron was the most represented variant (86%) in this cohort.

### Characteristics of patients at diagnosis

Patients’ characteristics at diagnosis are displayed in Table 1. The median age was 60 years old (interquartile range [IQR]: [51–71]), and 64% were men. Twenty-eight patients (28/53; 53%) were SOT recipients (two heart, 23 kidney, two lungs, one combined kidney and liver), while 42% had hematological malignancies (HMs) (22/53; including two of the SOT recipients for whom the HM was considered as the main underlying disease). The most represented HMs were non-Hodgkin lymphoma (12/22), chronic lymphocytic leukemia (3/22), and acute myeloid leukemia (2/22, including one patient with hematopoietic stem cell transplantation, HSCT). Other HMs included one case of multiple myeloma, one case of Waldenström’s macroglobulinemia, one case of myelodysplastic syndrome (with refractory cytopenia), one case of prolymphocytic leukemia, and one case of tricholeukocytic leukemia. Nine percent (5/53) had another type of immunosuppression. In SOT recipients, the median time from transplantation to SARS-CoV-2 diagnosis was 25 (3–96) months.

**TABLE 1:** Patients’ characteristics at diagnosis, according to the main underlying disease^*a*^

	Total (*n* = 53)	HM (*n* = 22)^*c*^	SOT without HM (*n* = 26)	Other IS (*n* = 5)^*b*^	*P*-value^*d*^
Age at diagnosis	60 [51–71]	66.5 [58–78]	57.5 [49–65]	41 [35–45]	**<0.01**
Men	34/53 (64%)	16/22 (73%)	14/26 (54%)	4/5 (80%)	0.34
Comorbidities					
- Hypertension	21/53 (40%)	6/22 (27%)	15/26 (58%)	0	**0.02**
- Body mass index ≥30	5/53 (9%)	2/22 (9%)	3/26 (12%)	0	1
- Diabetes mellitus	12/53 (23%)	3/22 (14%)	9/26 (35%)	0	0.19
SARS-CoV-2 variant					1
- Omicron	32/37 (86%)	12/13 (92%)	17/21 (81%)	3/3 (100%)	
- Delta	1/37 (3%)	0	1/21 (5%)	0	
- Alpha	4/37 (11%)	1/13 (8%)	3/21 (14%)	0	
Detection of SARS-CoV-2 RNA in serum	9/18 (50%)	6/10 (60%)	3/7 (43%)	0	0.64
IgG SARS-CoV-2 ⩾ 260 BAU/mL at diagnosis	14/36 (39%)	6/14 (43%)	8/20 (40%)	0	0.74
Severity of SARS-CoV-2 infection					
- Non-severe	36/53 (68%)	12/22 (55%)	20/26 (77%)	4/5 (80%)	0.22
- Severe	17/53 (32%)	10/22 (45%)	6/26 (23%)	1/5 (20%)	
Lesions on CT scan at diagnosis	28/34 (82%)	13/14 (93%)	13/16 (81%)	2/4 (50%)	0.14
- <10%	2/25 (8%)	0/12	2/11 (18%)	0	
- Mild (10–25%)	14/28 (50%)	5/13 (38%)	8/13 (62%)	1/2 (50%)	
- Moderate (25–50%)	9/28 (32%)	6/13 (46%)	2/13 (15%)	1/2 (50%)	
- Severe (50–75%)	1/28 (3%)	0	1/13 (8%)	0	
- Critical >75%	0	0	0	0	
Immunosuppressive treatment					
- IL12/23 inhibitors	2/49 (4%)	1/21 (5%)	0	1/5 (20%)	0.10
- Corticosteroids (equivalent prednisone 5 mg/day or higher, for over 3 months)	33/53 (62%)	7/22 (32%)	23/26 (88%)	3/5 (60%)	**<0.01**
- Mycophenolate mofetil	24/53 (45%)	0	24/26 (92%)	0	**<0.01**
- CD20/19 inhibitors	17/53 (32%)	14/22 (64%)	2/26 (8%)	1/5 (20%)	**<0.01**
- Venetoclax	7/53 (13%)	7/22 (32%)	0	0	**<0.01**
- BTK inhibitors	5/53 (9%)	5/22 (23%)	0	0	**0.03**
- PI3K inhibitors	1/53 (2%)	1/22 (5%)	0	0	0.51
- Belatacept	6/53 (11%)	0	6/26 (23%)	0	**0.04**
- Calcineurin inhibitors	23/53 (43%)	3/22 (14%)	20/26 (77%)	0	**<0.01**
- mTOR inhibitors	2/52 (4%)	1/22 (5%)	1/25 (4%)	0	1
- Other type of chemotherapy	13/53 (25%)	13/22 (59%)	0	0	**<0.01**

^*a*^ BTK, Bruton tyrosine kinase; HM, hematological malignancy; IL, interleukin; IS, immunosuppressant; PI3K, Phosphoinositide 3-kinase; SOT, solid organ transplantation; BAU/mL, binding antibody units per milliliter; mTOR, mammalian target of rapamycin.

^*b*^ Including 3 patients with HIV, 1 patient with primary immunodeficiency.

^*c*^ 2 patients with hematological malignancy (HM) were also solid organ transplant recipients (HM was retained as the main underlying disease).

^*d*^ Bolding indicates that the *P*-value is <0.05 (statistically signifiant).

Among the 53 patients included, the data on prophylaxis received was not available for eight of them. Among the available data, 16 received no prophylaxis, whether vaccine or monoclonal antibodies. Among the 29 patients who received prophylaxis, three received one dose of vaccine, seven received two doses, 15 received three doses, and four received four doses of vaccine. Among those who received two doses, two also received tixagevimab/cilgavimab as a prophylaxis, while 10 of those who received three doses received monoclonal antibodies prophylactically (seven tixagevimab/cilgavimab and three casirivimab/imdevimab), and one of those who received four doses also received tixagevimab/cilgavimab prophylactically. One patient received only prophylaxis with tixagevimab/cilgavimab. The most represented immunosuppressive treatments were corticosteroids (62%), mycophenolate mofetil (45%), calcineurin inhibitors (43%), and CD19/20 inhibitors (32%). Sixty-one percent of the patients had anti-SARS-CoV-2 IgG levels <260 BAU/mL at diagnosis (i.e., unvaccinated or weak responders to vaccination or previous infection).

Patients had frequent comorbidities, including 40% with hypertension and 23% with diabetes.

### Clinical evolution and complications

Forty-eight patients were hospitalized during their SARS-CoV-2 infection (Table 2): 13 were admitted to the day hospital for upper or lower respiratory tract infection that did not require oxygen therapy, and 35 were hospitalized in conventional units. Among them, 29 had pneumonia; six presented alterations of the general status, including three with weight loss, two with prolonged fever, and two with acute kidney injury; and six were hospitalized because of clinical symptoms and underlying diseases. Among the 28 patients for whom this information was available, the median time from diagnosis to hospitalization was 46 days (15–94). Seventeen of the hospitalized patients had severe infections, defined as requiring hospitalization with >3L oxygen and/or corticosteroids or tocilizumab, including 10 patients with HM (10/17; 59%). Furthermore, most of the patients (28/34; 82%) for whom chest CT scan was available had lesions compatible with SARS-CoV-2 infection at diagnosis, mainly mild (affecting 10% to 25% of the lungs).

**TABLE 2:** Complications of SARS-CoV-2 infection, according to the main underlying disease^*a*^

	Total (*n* = 53)	HM (*n* = 22)	SOT (*n* = 26)	Other IS (*n* = 5)	*P*-value
IMIs	9/52 (19%)	4/22 (18%)	5/26 (19%)	0/4 (0%)	1
Hospitalization	48/52 (92%)	19/21 (90%)	24/26 (92%)	5/5 (100%)	0.71
6-month mortality	6/53 (11%)	2/22 (9%)	3/26 (12%)	1/5 (20%)	0.68
1-year mortality	10/53 (19%)	5/22 (23%)	4/26 (15%)	1/5 (20%)	0.88

^*a*^ HM, hematological malignancy; IMI, invasive mold infections; IS, immunosuppressant; SOT, solid organ transplantation.

**Fig 1:**
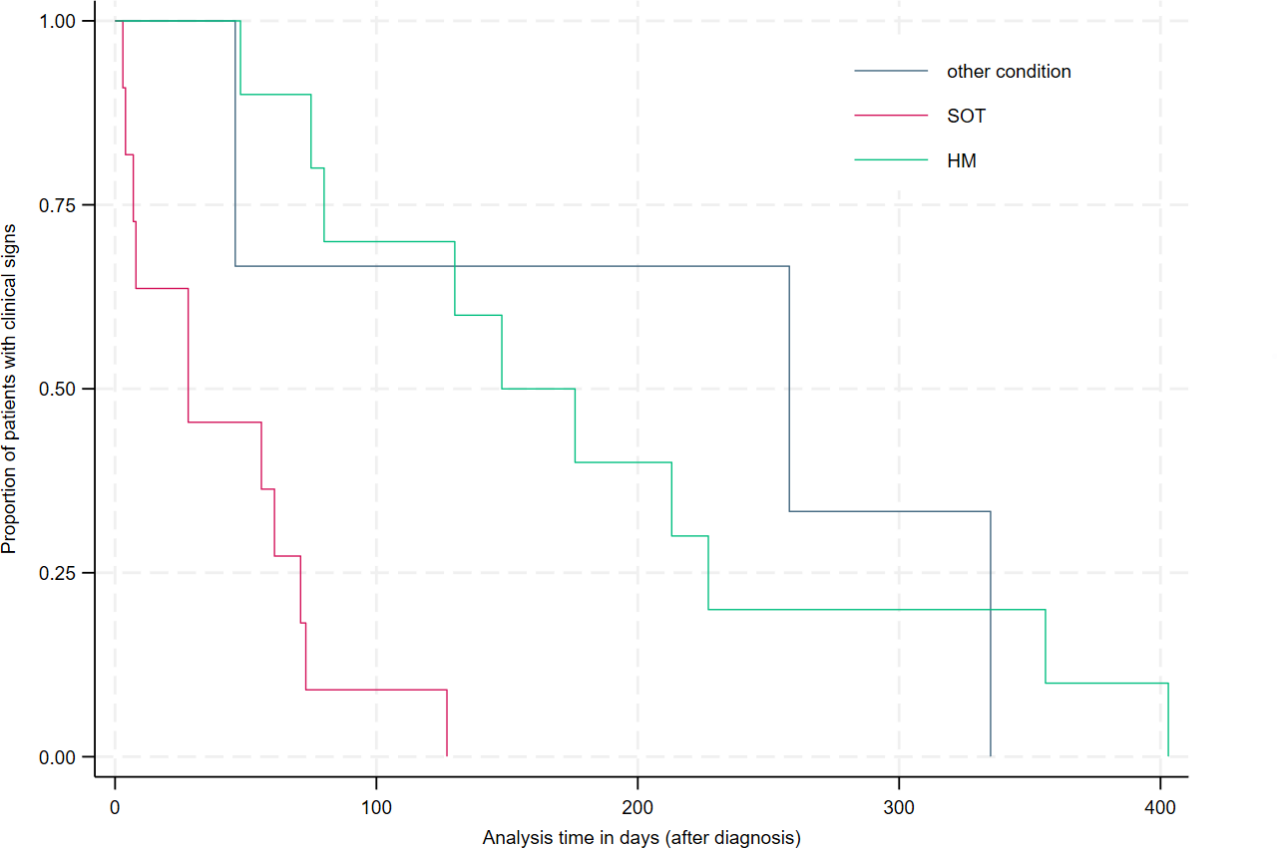
Time to clinical cure after diagnosis, according to the main underlying disease, univariate analysis

During follow-up, 41 of 47 patients (87%) with available data achieved clinical cure. The median time to clinical cure was significantly longer in patients with severe disease than in those with non-severe infection (213 [148–258] days vs. 71 [28–130] days; *P* = 0.04). SOT recipients reached clinical cure faster than patients with HM (28 [7–71] vs. 162 [80–227] days; *P* < 0.01), even after adjusting for disease severity (Fig. 1). Patients receiving CD20/19 inhibitors had a significantly longer duration of symptoms than those without (162 days [103.5–281] vs. 37 days [7.5–66]; *P* < 0.01), even after adjusting for disease severity. Having anti-SARS-CoV-2 IgG levels <260 BAU/mL at diagnosis did not significantly affect time to clinical cure or time to CT scan normalization (*P* = 0.68 and 0.07, respectively). Seventeen percent of patients (*n* = 9) experienced IMIs, including eight cases of aspergillosis and one coinfection with *Aspergillus* spp. and *Rhizopus* spp. (Table 2). IMIs were more likely to occur in patients with severe SARS-CoV-2 (41% vs. 6%, *P* = 0.03). IMIs tended to be associated with increased six-month and one-year mortality (*P* = 0.06). In these patients with persistent viral shedding, one-year mortality was significantly associated with severe SARS-CoV-2 (*P* = 0.04), defined as requiring the use of dexamethasone and/or tocilizumab.

### Characteristics of the treatment received and impact on clinico-radiological outcomes

Seventy-five percent (40/53) of patients received at least one anti-SARS-CoV-2 treatment. The median time between diagnosis and treatment initiation was three days (1–42). Among the 24 patients who received more than one treatment, 41% (10/24) received all lines of treatments over a 21-day period, while the other 59% were treated sequentially, over a longer period (Fig. S1).

First-line therapies are detailed in Table 3. Patients could have received direct antiviral(s) alone, mab alone, plasma alone, combination of antivirals + mab, or combination of antivirals + plasma. The duration of treatment with nirmatrelvir/ritonavir when involved in first-line treatment was always five days, while the median duration of remdesivir was three days (3–5). Statistical comparison of outcomes between these groups was not performed due to small sample size in each treatment strategy. However, Table S1 provides descriptive data on time to clinical cure, viral clearance, and CT normalization according to the first-line treatment strategy. Overall, patients treated with direct antivirals seemed to have better outcomes. Thus, analysis was performed to compare outcomes between treatment strategy involving direct antivirals or those that did not. There was no significant impact of first-line treatment with direct antivirals on the time to clinical cure or time to radiological normalization, even after adjustment for confounding variables (i.e., disease severity and underlying condition). Among the hospitalized patients, 12 had previously received outpatient treatment with direct-acting antiviral monotherapy (*n* = 6), monoclonal antibodies (mAbs) (*n* = 5), or a combination of both (*n* = 1). Receiving a first-line treatment involving plasma (*n* = 12) did not have a significant effect on time to clinical cure (median 68 [28–194] days without plasma vs. 111 [72–188] days with plasma) or time to CT scan normalization (median 126 [83–198] days without plasma vs. 321 [127–452] days with plasma), compared to other first-line treatment strategies, after adjusting on disease severity, main underlying disease, and time before treatment initiation (respectively *P* = 0.11; and *P* = 0.86). Eight of the patients treated with plasmatherapy had IMIs. Data and outcomes for patients in whom plasma was used as first-line treatment or after are detailed in Table S3.

**TABLE 3:** First-line treatments received, according to the main underlying disease (*n* = 40/53)^*e*^

	Total (*n* = 40)	HM (*n* = 18)	SOT (*n* = 19)	Other IS (*n* = 3)	*P*-value
First-line treatment					0.49
Antiviral monotherapy^*a*^	10/40 (25%)	7/18 (39%)	3/19 (16%)	0	
Plasmatherapy alone	7/40 (18%)	4/18 (22%)	3/19 (16%)	0	
mAb(s) exclusively^*b*^	17/40 (43%)	5/18 (28%)	9/19 (47%)	3/3 (100%)	
Antiviral therapy + mAb^*c*^	1/40 (2%)	0	1/19 (5%)	0	
Antiviral therapy + plasma^*d*^	5/40 (12%)	2/18 (11%)	3/19 (16%)	0	

^*a*^ Antiviral monotherapy of remdesivir in 3 patients (8%) and of nirmatrelvir/ritonavir in 7 patients (18%).

^*b*^ Monotherapy of mAb(s): 6 patients received sotrovimab monotherapy (15%), 8 patients received tixagevimab/cilgavimab (20%), and 3 patients received casirivimab/imdevimab (8%).

^*c*^ Antiviral therapy + mAb = remdesivir plus tixagevimab/cilgavimab.

^*d*^ Antiviral therapy + plasma: all 5 patients received a combination of remdesivir and plasma (none had plasma plus nirmatrelvir/ritonavir).

^*e*^ HM, hematological malignancy; mAb, monoclonal antibody; SOT, solid organ transplantation.

### Virus dynamics during persistent infection

**Fig 2:**
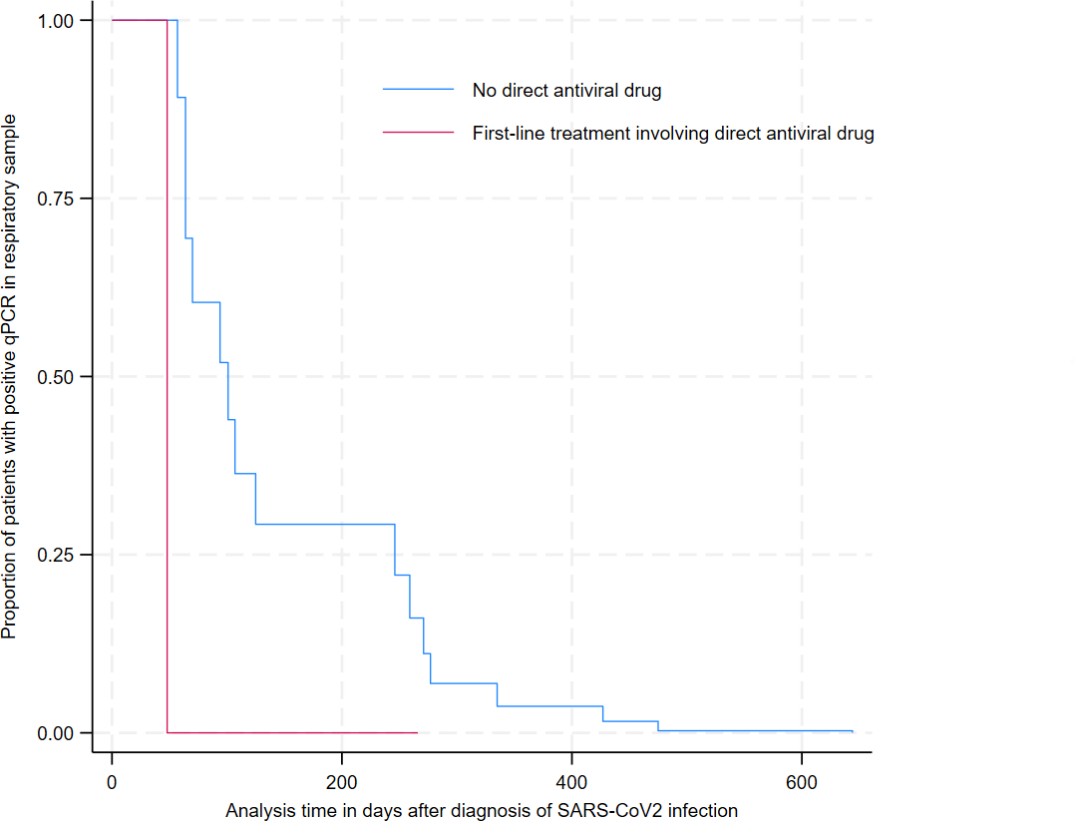
Time to viral clearance according to first-line treatment, after adjusting for disease severity, main underlying condition, and time before first treatment initiation

All patients exhibited persistent viral shedding >8 weeks, and 34 (65%) eventually achieved viral clearance during follow-up. The median time before viral clearance in patients who achieved viral cure was 125 days (94–266) (Table 3). Notably, the time to viral clearance was not significantly impacted by disease severity, baseline anti-SARS-CoV-2 IgG levels <260 BAU/mL (*P* = 0.07), or underlying condition (111 [93–271] days in SOT recipients vs. 142 [70–266] days in HM patients, *P* = 0.67) (Table S2). However, first-line treatment involving direct antiviral drugs significantly accelerated viral clearance after adjustment for confounding factors, with a median time of 107 days ([51–118], mean 111 days), compared to 183 days ([97–277], mean 224 days) in patients who did not receive remdesivir or nirmatrelvir/ritonavir (*P* = 0.04) (Fig. 2). In the 12 patients who received a first-line treatment involving plasmatherapy, the median time to viral clearance was 266 [114–427] days (vs. 115 [70–259] days without plasma; *P* = 0.14 after adjusting on disease severity, main underlying disease, and time before treatment initiation).

**Fig 3:**
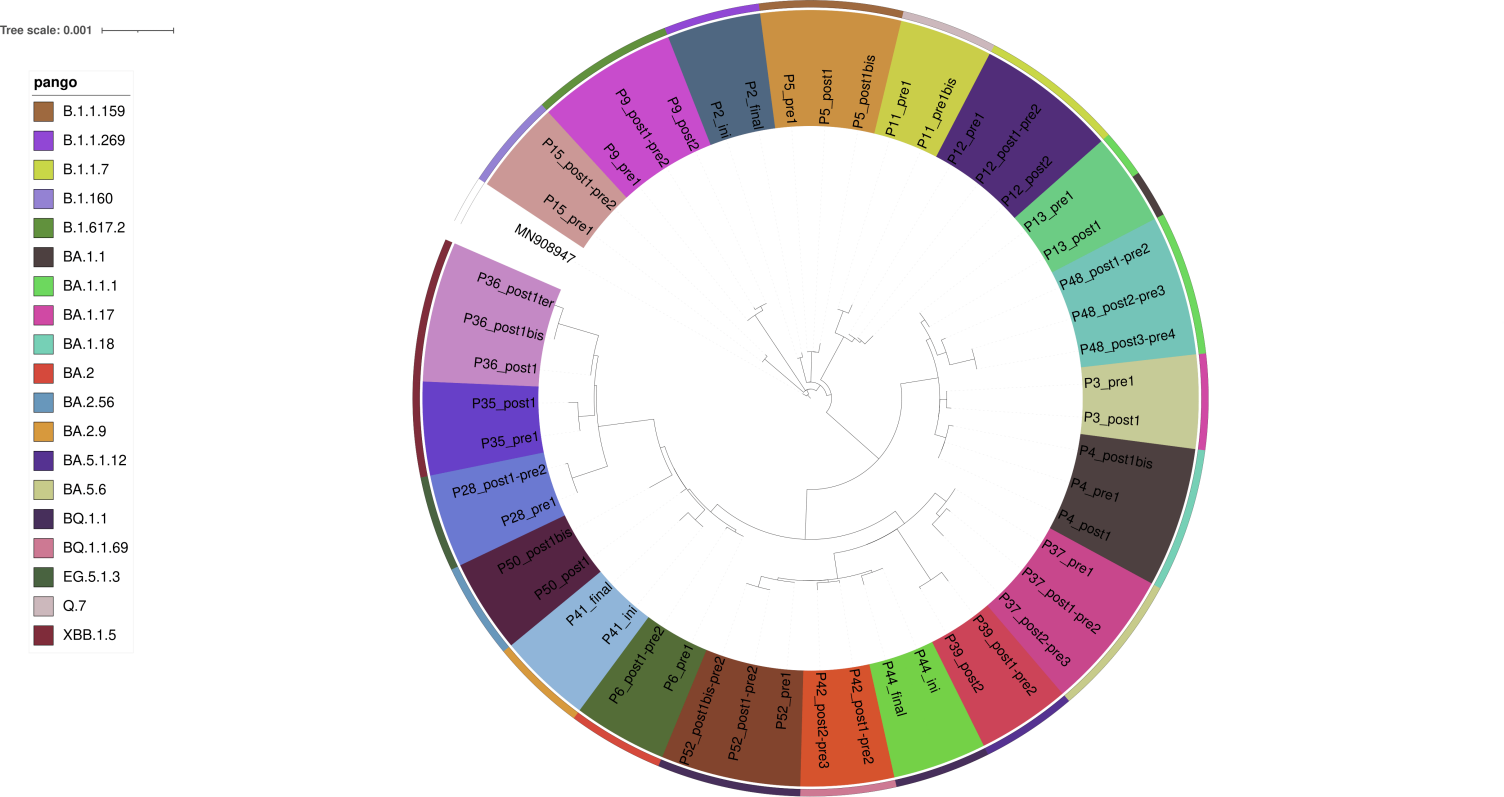
Phylogenetic tree of variant responsible for persistent viral shedding (>8 weeks) in immunocompromised patients. This is a phylogenetic tree rooted in the Wuhan reference genome (MN908927), where each branch represents evolutionary distances and nucleotide substitutions. Most patients’ samples have closely related branches, with later samples showing longer branches, indicating mutation accumulation over time. The tree helps differentiate persistent infections from reinfections. Notably, all patients’ samples were classified under the same viral variant lineage, except for patient P13, whose pre- and post-treatment samples were assigned slightly different lineages due to lower post-treatment sequence resolution rather than a true variant change.

**Fig 4:**
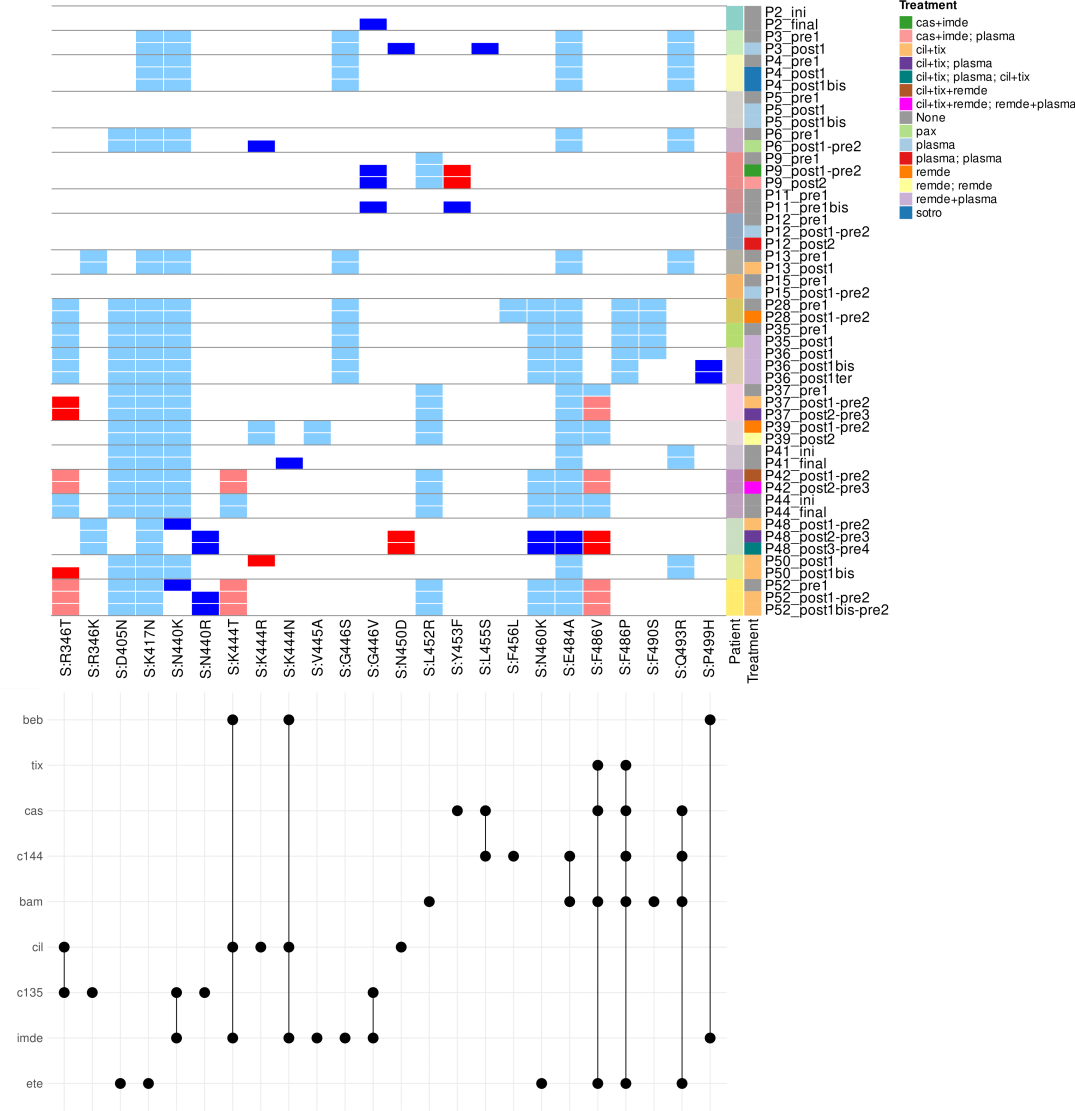
Mutations linked to susceptibility or resistance to treatments in major variant populations. Upper part of the figure: mutations (shown by columns) for each patient across time and according to treatment. Light blue: Initial presence of a mutation in a patient, which does not affect any of the treatments received thereafter. Dark blue: Acquisition or loss of a relevant mutation during time of persistent infection, with no impact on the sensitivity of SARS-CoV-2 to any of the treatments received. Light red: Initial presence of a mutation that affects the sensitivity of SARS-CoV-2 to one or more treatments received thereafter. Dark red: Emergence of a relevant mutation during persistent infection that impacts the sensitivity of SARS-CoV-2 to one or more treatments received. Lower part of the figure: molecules affected by each mutation detected. For each mutation (columns), treatments affected between sotrovimab (sotro), tixagevimab (tix), cilgavimab (cil), casirivimab (cas), imdevimab (imde), remdesivir (remde), nirmatrelvir (nirm), ritonavir (rito), bebtelovimab (beb), monoclonal antibody c144 (c144), monoclonal antibody c135 (c135), bamlanivimab (bam), and etesevimab (ete) are shown by a black spot.

A total of 50 samples from 21 patients were selected for further analysis, as outlined in the Methods section and Fig. S2 (“Sample Preparation and Sequencing” and “Mutation and Resistance Analysis”). Phylogenetic analysis of samples before and after anti-SARS-CoV-2 treatment revealed that the majority of patients had closely related virus strains, with later samples showing longer branches, indicating mutation accumulation over time (Fig. 3). The emergence of mutations in major variant populations is reported in Fig. 4. Pre-treatment, 13 out of 17 patients for whom pre-treatment sequencing data were available (76%) had already exhibited resistance mutations to anti-SARS-CoV-2 treatments. Among them, only two (15%) had received mAbs as prophylaxis, and eight (62%) had been vaccinated. During persistent viral shedding, 11/21 patients developed mutations, including three (patients 5, 12, and 15) without any prior treatment pressure (even no mAb prophylaxis or vaccine exposure). However, the majority of these patients (8/11; 73%) developed resistance mutations after receiving curative treatments (as opposed to mAb prophylaxis or vaccination). Specifically, four patients developed mutations directly conferring resistance to previously administered treatments, all mAb-based. Mutations included Y453F (resistance to casirivimab, after exposure to casirivimab/imdevimab in patient 9), R346T (resistance to cilgavimab and c135, after exposure to tixagevimab/cilgavimab in patient 37), N450D and F486V (resistance to tixagevimab, casirivimab, bamlanivimab, and etesevimab, after exposure to tixagevimab/cilgavimab and plasmatherapy in patient 48), and K444R (resistance to cilgavimab, after exposure to tixagevimab/cilgavimab in patient 50). Among the six patients treated with curative mAbs, only two did not develop resistance mutations. In patients receiving plasmatherapy, 27% (3/11) developed mutations on the spike protein conferring resistance to mAbs, including two previously exposed to mAbs (curative tixagevimab/cilgavimab in patient 48, prophylactic mAbs before infection in patient 36). Interestingly, the third patient (patient 3) developed N450D and L455S mutations after plasmatherapy despite never having received mAbs or vaccination. Among the five patients treated with remdesivir, four did not develop resistance mutations. One patient (patient 36) developed a mutation on the spike protein conferring resistance to mAbs (P499H) after receiving remdesivir plus plasmatherapy, as well as prior prophylactic mAbs. In the case of a vaccinated patient treated with nirmatrelvir/ritonavir, resistance to cilgavimab emerged after monotherapy, despite no prior mAbs exposure (patient 6).

Overall, 19 patients received monoclonal antibodies (mAbs) during their infection, including 17 as first-line treatment. Of these, pre-treatment sequencing data were available for eight patients (not known to the clinician at the time of treatment initiation). Notably, four of them (50%) received curative mAbs despite the presence of preexisting mutations known to reduce SARS-CoV-2 sensitivity to these antibodies (patients 37, 42, 48, and 52). Minor variant analysis revealed treatment-resistant mutations in five patients, including two who developed resistance to mAbs (Fig. S3). However, the small sample size of this analysis limits any conclusions regarding the impact of these mutations on clinical outcomes or viral clearance.

## DISCUSSION

ICPs with persistent SARS-CoV-2 viral shedding (>8 weeks) represent a highly vulnerable population burdened by significant comorbidities (mostly hypertension and diabetes), low anti-SARS-CoV-2 antibodies at diagnosis (nearly two-thirds had IgG levels <260 BAU/mL), and severe complications including hospitalization (91%) and the need for oxygen therapy and corticosteroid (one-third of the cases). This result reinforces previous findings (4). Almost one-fifth of the patients (17%) developed invasive mold infections without necessarily having been hospitalized in an intensive care unit, which is comparable to a previous report (20%) (6).

First-line treatments involving direct antiviral therapies (i.e., remdesivir and/or nirmatrelvir/ritonavir) were associated with faster viral clearance in ICPs compared to treatments involving mAbs or plasmatherapy. Furthermore, neither plasmatherapy nor mAbs demonstrated superiority in reducing the time to viral clearance, even in patients without prior resistance mutations to the mAbs used (mainly in the “pre-Omicron” period). Consistently, recent studies have shown that remdesivir is associated with reduced mortality in hospitalized ICPs, particularly those with HMs or SOT (21). Additionally, studies have suggested that prolonged or combined courses of direct antivirals could be beneficial for ICPs (12, 13, 22, 23). Thus, it seems crucial to intensify the therapeutic approach for profoundly immunosuppressed patients (particularly SOT and HM), going beyond the guidelines which propose short, early antiviral treatment in severe or comorbid patients. Indeed, a prolonged, systematic treatment regimen with direct antiviral(s) in at-risk patients, repeated in cases of persistence, appears to be warranted.

Recent reports also indicate that some ICPs with persistent SARS-CoV-2 infections may exhibit reduced responses to direct antiviral therapies, such as remdesivir and nirmatrelvir/ritonavir, due to the emergence of resistance mutations (2, 24). In our series, we did not identify any of these specific resistance mutations in major SARS-CoV-2 variants, although 85% of the patients whose samples were analyzed showed mutations conferring resistance to mAb(s)—either preexisting or acquired during the course of infection. It has been shown that the emergence of resistance mutations is not necessarily driven by treatment-induced selective pressure. Indeed, SARS-CoV-2 evolution is largely shaped by immune escape, and some resistance-associated mutations have become lineage-defining. However, resistances to mAb(s) particularly emerged in patients pre-exposed to mAbs, but also in one patient treated with plasmatherapy (1/11; 9%) who developed mutations on spike protein conferring resistance to mAbs (25). These types of mutations were observed in major variants of only one out of five patients receiving remdesivir, but this patient had also been pre-exposed to prophylactic mAbs and curative plasmatherapy (26).

This retrospective study has several limitations. First, it focuses on a highly specific population (patients with persistent viral shedding >8 weeks) and includes a heterogeneous mix of underlying conditions (e.g., HM and SOT) and treatment regimens. These factors may limit the generalizability of the findings, and the low number of cases limits statistical power. However, we have established that remdesivir and nirmatrelvir/ritonavir were the most effective treatments for these hospitalized immunocompromised patients with persistent viral shedding, both in terms of viral clearance efficacy and genetic barrier. This is especially pertinent in the current context, where the Omicron variant has developed resistance to all available therapeutic monoclonal antibodies. Furthermore, even if new monoclonal antibodies theoretically effective against Omicron are developed, their superiority over direct antivirals will need to be proven (27). It will also be crucial to closely monitor mutations in immunocompromised patients before and during such treatments or plasmatherapy. Furthermore, these findings should be taken into account when considering therapeutic recommendations for any future viral epidemic or pandemic: monoclonal antibody monotherapy appears to be a riskier option compared to combined or repeated direct antiviral therapies.

### Conclusion

The findings of this study emphasize that the use of direct antiviral therapies (remdesivir and nirmatrelvir/ritonavir) is the most effective treatment in immunocompromised patients (ICPs), reducing the time to SARS-CoV-2 viral clearance, which has been shown to be associated with invasive mold infections. Moreover, their genetic barriers are adequate.

## SUPPLEMENTAL MATERIAL

Fig. S1 aac.00658-25-s0001.tiff Time to clinical cure and viral clearance, and time to treatment initiation.

Fig. S2 aac.00658-25-s0002.tiff Flow chart selection of nasopharyngeal sample for sequencing analysis.

Fig. S3 aac.00658-25-s0003.tiff Mutations in minor variants associated with treatment response.

Supplemental material aac.00658-25-s0004.docx Legends for all supplemental figures and tables.

Table S1 aac.00658-25-s0005.docx Evolution of SARS-CoV-2 infection, according to the first-line treatment.

Table S2 aac.00658-25-s0006.docx Evolution of SARS-CoV-2 infection, according to the main underlying disease.

Table S3 aac.00658-25-s0007.docx Outcomes in patients who received plasmatherapy.

ASM does not own the copyrights to Supplemental Material that may be linked to, or accessed through, an article. The authors have granted ASM a non-exclusive, world-wide license to publish the Supplemental Material files. Please contact the corresponding author directly for reuse.
