# Supplementary figures and images for: Immunocompromised patients with persistent SARS-CoV-2 viral shedding ≥8 weeks, clinical outcomes, and virological dynamics: a retrospective multicenter cohort study, 2020–2024
Source: Antimicrob Agents Chemother. 2025 Sep 26;69(11):e00658-25. doi: 10.1128/aac.00658-25 (PMC12587602; doi:10.1128/aac.00658-25)

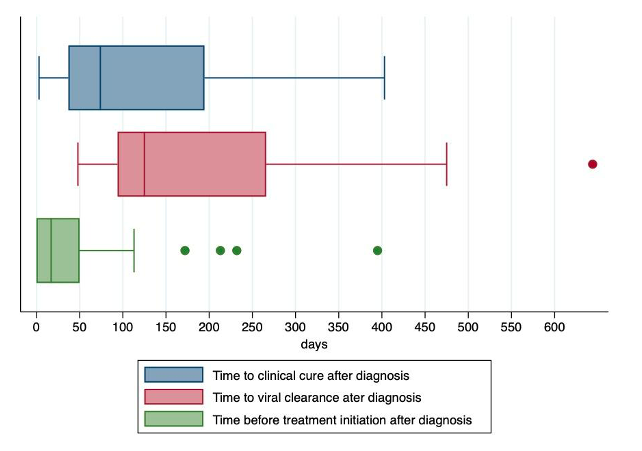

Supplement: Fig. S1 — Time to clinical cure and viral clearance, and time to treatment initiation. [file aac.00658-25-s0001.tiff]

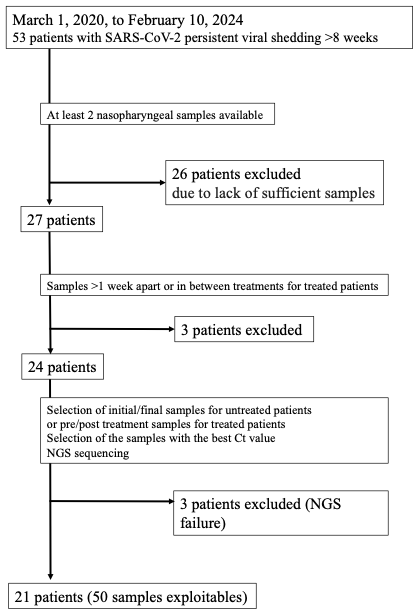

Supplement: Fig. S2 — Flow chart selection of nasopharyngeal sample for sequencing analysis. [file aac.00658-25-s0002.tiff]

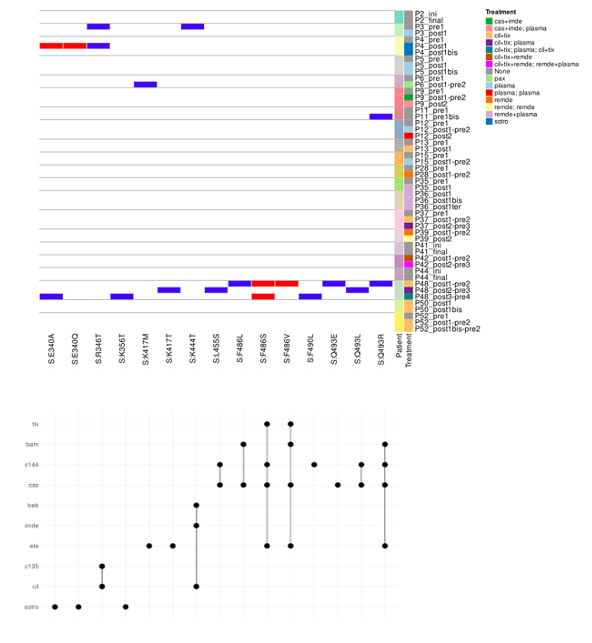

Supplement: Fig. S3 — Mutations in minor variants associated with treatment response. [file aac.00658-25-s0003.tiff]
