# Supplementary material for: Immunocompromised patients with persistent SARS-CoV-2 viral shedding ≥8 weeks, clinical outcomes, and virological dynamics: a retrospective multicenter cohort study, 2020–2024
Source: Antimicrob Agents Chemother. 2025 Sep 26;69(11):e00658-25. doi: 10.1128/aac.00658-25 (PMC12587602; doi:10.1128/aac.00658-25)
Supplement: Supplemental material — Legends for all supplemental figures and tables. [file aac.00658-25-s0004.docx]

**SUPPLEMENTARY DATA**

**Supplementary Table 1. Evolution of SARS-CoV-2 infection, according to the first line treatment**

HM: hematologial malignancy; IS: immunosuppressant; SOT: solid organ transplantation.

Time unit: median in days [interquartile range 25-75]

**Supplementary Table 2. Evolution of SARS-CoV-2 infection, according to the main underlying disease**

HM: hematologial malignancy; IS: immunosuppressant; SOT: solid organ transplantation.

Time unit: median in days [interquartile range 25-75]

**Supplementary Table 3. Outcomes in patients who received plasmatherapy**

**Supplementary Figure 1**. **Time to clinical cure and viral clearance, and time to treatment initiation**

**Supplementary Figure 2.** Flow chart selection of nasopharyngeal sample for Sequencing analysis

**Supplementary Figure 3. Mutations in minor variants associated with treatment response**
