# Supplementary material for: Immunocompromised patients with persistent SARS-CoV-2 viral shedding ≥8 weeks, clinical outcomes, and virological dynamics: a retrospective multicenter cohort study, 2020–2024
Source: Antimicrob Agents Chemother. 2025 Sep 26;69(11):e00658-25. doi: 10.1128/aac.00658-25 (PMC12587602; doi:10.1128/aac.00658-25)
Supplement: Table S1 — Evolution of SARS-CoV-2 infection, according to the first-line treatment. [file aac.00658-25-s0005.docx]

**Supplementary Table 1. Evolution of SARS-CoV-2 infection, according to the first line treatment**

|  | **No treatment (n=13)** | **First line treatment with mAbs alone (n=17)** | **First line treatment with direct antivirals alone (n=10)** | **First line treatment with plasma alone (n=7)** | **First line treatment with plasma + direct antiviral (n=5)** | **First line treatment with mAb + direct antiviral (n=1)** |
| --- | --- | --- | --- | --- | --- | --- |
| **Time before clinical cure after diagnosis*** | 7 [4-258] (mean 126) | 56 [28-213]  (mean 107) | 104 [62-153] (mean 133) | 73 [71-148] (mean 97) | 227 [227-227]  (mean 227) | - |
| **Time before viral clearance after diagnosis*** | 181 [111-261]  (mean 211) | 107 [70-259]  (mean 170) | 51 [48-70]  (mean 56) | 349 [259-536]  (mean 397) | 114 [107-266]  (mean 162) | 118 [118-118]  (mean 118) |
| **Time before CT normalization after diagnosis*** | 83 [76-123]  (mean 94) | 255 [136-320]  (mean 254) | 113 [48-134]  (mean 108) | 290 [127-452]  (mean 290) | 321 [321-321]  (mean 321) | - |

HM: hematologial malignancy; IS: immunosuppressant; SOT: solid organ transplantation.

Time unit: median in days [interquartile range 25-75]
