# Supplementary material for: Immunocompromised patients with persistent SARS-CoV-2 viral shedding ≥8 weeks, clinical outcomes, and virological dynamics: a retrospective multicenter cohort study, 2020–2024
Source: Antimicrob Agents Chemother. 2025 Sep 26;69(11):e00658-25. doi: 10.1128/aac.00658-25 (PMC12587602; doi:10.1128/aac.00658-25)
Supplement: Table S2 — Evolution of SARS-CoV-2 infection, according to the main underlying disease. [file aac.00658-25-s0006.docx]

**Supplementary Table 2. Evolution of SARS-CoV-2 infection, according to the main underlying disease**

|  | **Total (n=53)** | **HM (n=22)** | **SOT (n=26)** | **Other IS (n=5)** | **p-value** |
| --- | --- | --- | --- | --- | --- |
| **Time before clinical cure after diagnosis** | 74 [37-195] | 162 [80-227] | 28 [7-71] | 258 [46-335] | **<0.01** |
| **Time before viral clearance after diagnosis** | 125 [94-266] | 142 [70-266] | 111 [93-271] | 183 [153-260] | 0.67 |
| **Time before CT-scan normalization after diagnosis** | 129 [98-255] | 132 [98-198] | 126.5 [98-344] | - | 0.79 |

HM: hematologial malignancy; IS: immunosuppressant; SOT: solid organ transplantation.

Time unit: median in days [interquartile range 25-75]
