# Supplementary material for: Immunocompromised patients with persistent SARS-CoV-2 viral shedding ≥8 weeks, clinical outcomes, and virological dynamics: a retrospective multicenter cohort study, 2020–2024
Source: Antimicrob Agents Chemother. 2025 Sep 26;69(11):e00658-25. doi: 10.1128/aac.00658-25 (PMC12587602; doi:10.1128/aac.00658-25)
Supplement: Table S3 — Outcomes in patients who received plasmatherapy. [file aac.00658-25-s0007.docx]

**Supplementary Table 3.** **Outcomes in patients who received plasmatherapy**

| **Patient number** | **Age/ sex** | **Main underlying condition** | **Plasma in first-line treatment** | **Treatment before Plasmatherapy** | **Delay between positive sample and plasma (in days)** | **Delay between initial clinical symptom and plasmatherapy (in days)** | **Time before clinical recovery after plasmatherapy (days)** | **Time before viral cure after plasmatherapy (days)** | **IMI** |
| --- | --- | --- | --- | --- | --- | --- | --- | --- | --- |
| 3 | 28/F | SOT (kidney) | YES | None | 64 | 76 | 7 | 363 | 0 |
| 5 | 45/F | SOT (kidney) | YES | None | 48 | 48 | 25 | 223 | 0 |
| 6 | 44/F | SOT (kidney) | NO | nirmatrelvir/ ritonavir | 112 | 37 | 15 | No negative qPCR during follow-up | 0 |
| 9 | 71/F | SOT (kidney) | NO | mAbs | 38 | 38 | 23 | 26 | 1 |
| 12 | 80/F | HM | YES | None | 2 | 19 | 146 | NA | 1 |
| 15 | 61/M | HM (+SOT) | YES | None | 10 | 13 | 106 | No negative qPCR during follow-up | 0 |
| 16 | 61/M | HM (+SOT) | NO | tixagévimab/ cilgavimab | 27 | 29 | 186 | No negative qPCR during follow-up | 1 |
| 19 | 41 | other | NO | mAbs | 64 | 64 | No clinical recovery during follow-up | 61 | 0 |
| 22 | 63/M | HM (DLBCL) | YES | None | NA | 84 | No clinical recovery during follow-up (partial clinical improvement) | NA | 0 |
| 25 | 55/F | SOT (lung) | YES | None | 203 | 203 | No clinical recovery during follow-up | 43 | 1 |
| 26 | 87/F | HM (CLL) | NO | remdesivir | 30 | 41 | 373 | No negative qPCR during follow-up | 0 |
| 27 | 58/M | HM (Mantle lymphoma) | YES (+remdesivir) | None | 223 | 225 | 4 | 43 | 0 |
| 28 | 62/M | HM (Waldenstrom) | NO | remdesivir | 30 | 37 | 5 | No negative qPCR during follow-up | 0 |
| 31 | 52/M | HM (Follicular lymphoma) | NO | nirmatrelvir/ritonavir | 175 | 177 | 1 | No negative qPCR during follow-up | 0 |
| 32 | 57 | HM | NO | remdesivir | 64 | 64 | 11 | No negative qPCR during follow-up | 0 |
| 33 | 53/M | SOT (kidney) | YES (+remdesivir) | None | NA | 669 | NA | No negative qPCR during follow-up | 0 |
| 34 | 58 | HM | YES (+remdesivir) | None | 81 | 81 | 33 | 33 | 0 |
| 35 | 77/M | SOT (kidney) | YES (+remdesivir) | None | NA | 25 | NA | No negative qPCR during follow-up | 1 |
| 36 | 73 | SOT | YES (+remdesivir) | None | NA | NA | NA | NA | 1 |
| 37 | 77 | HM | NO | mAbs | 62 | 62 | 8 | 10 | 1 |
| 38 | 76 | HM | NO | nirmatrelvir /ritonavir | 68 | 68 | 12 | 2 | 0 |
| 42 | 60/F | SOT (heart) | NO | remdesivir  tixagévimab/ cilgavimab | 16 | 5 | 102 | 102 | 0 |
| 48 | 79/M | HM (prolymphocytic leukemia) | NO | sotrovimab | 91 | 93 | NA | 168 | 0 |
| 52 | 78/F | SOT (kidney) | NO | mAbs | 116 | 118 | No clinical recovery during follow-up | NA | 1 |
| 53 | 70/M | HM (CLL) | YES | None | 24 | 24 | 17 | No negative qPCR during follow-up | 0 |
